# The Role of Encephaloduroarteriosynangiosis in Moyamoya Disease: A Consecutive Case Series From Pakistan

**DOI:** 10.7759/cureus.83665

**Published:** 2025-05-07

**Authors:** Zubair M Khan, Sumira Kiran, Khawar Anwar, Sundas Irshad, Raahim A Bashir, Ch. Arslan Ahmad, Manal Khan, Maksalmina Reshtin, Haseeb Mehmood Qadri, Asif Bashir

**Affiliations:** 1 Neurological Surgery, Punjab Institute of Neurosciences, Lahore, PAK; 2 General Surgery, Lahore General Hospital, Lahore, PAK

**Keywords:** cerebral angiography, cerebral revascularization, hemorrhagic stroke, low- to middle-income countries, moyamoya disease (mmd), pakistan, pediatrics

## Abstract

Background

Moyamoya disease is a rare cerebrovascular disorder characterized by the occlusion of cerebral arteries due to stenosis. Although the disease lacks a specific treatment approach, various surgical options have been discovered to address ischemic issues, including indirect bypass, direct bypass, and their combination, which aim to enhance cerebral blood flow. Indirect revascularization via encephaloduroarteriosynangiosis (EDAS) is deemed effective in preventing additional strokes, although its use for preventing future hemorrhagic stroke in Moyamoya disease remains a subject of controversy.

Objective

The aim of this study was to evaluate the clinical outcomes of EDAS in hemorrhagic Moyamoya disease.

Methodology

This retrospective cohort study included eight consecutive patients with hemorrhagic Moyamoya disease who underwent indirect revascularization via EDAS from January 2019 to March 2024 at the Department of Neurosurgery, Punjab Institute of Neurosciences, Lahore, Pakistan. Of these eight patients, one patient was excluded owing to the non-availability of follow-up data. The data were collected in October 2024 by reviewing patient demographics, clinical characteristics, imaging findings, hematological parameters, treatment options, treatment outcomes, and follow-up data from hospital records.

Results

The mean age of study participants was 14.7±6.8 years. The mean preoperative Glasgow coma scale (GCS) score and the modified Rankin scale (mRS) score were 12.8±2.3 and 2.3±1.4, respectively. Following EDAS, four (57.1%) patients developed collateral circulation with grade A, whereas three (42.8%) patients had grade B according to the Matsushima grading system. The mean postoperative GCS score was 14.0±1.4, whereas the mean postoperative mRS score was 1.3±1.2. Only two patients developed postoperative ischemic symptoms, while none of them developed recurrent intracranial hemorrhage.

Conclusion

Appreciating the small size of the study, we recommend the effective utilization of EDAS in preventing strokes and improving functional outcomes in patients with hemorrhagic Moyamoya disease. The favorable role of EDAS is justified due to the lack of recurrent intracranial hemorrhage in our patients during the follow-up period.

## Introduction

Moyamoya disease (MMD) is an infrequently reported progressive vaso-occlusive condition with an unknown cause [1]. It is identified by the gradual narrowing of the terminal segments of internal carotid arteries on both sides, as well as the primary branches of the anterior and middle cerebral arteries, and is linked to collateral vessels at the brain's base (“moyamoya” vessels). When similar clinical symptoms are linked to an underlying condition, it is known as moyamoya syndrome. Nevertheless, due to the reliance on angiographic findings for the diagnostic criteria of this condition, it is highly advised to refrain from using the term “moyamoya syndrome” [2]. Pathological observations from autopsy cases, regardless of age, indicate the involvement of the distal portion of the carotid arteries and the proximal segments of the anterior and middle cerebral arteries. The arteries exhibit significant intimal thickening with twisted and duplicated internal elastic lamina, along with possible lipid accumulation in the intima. Notably, there is an absence of inflammatory indications and atheromatous plaque [3].

Initially believed to be predominantly found in Japan, cases of this disease have now been documented worldwide [4]. However, most reported cases still originate from Asia and other non-Caucasian areas [4]. Encephaloduroarteriosynangiosis (EDAS) is an indirect revascularization technique used in MMD to develop collaterals and bypass Moyamoya vasculature. In this technique, a single segment of the scalp artery is transposed to the brain's pial surface, which promotes collateral development via angiogenesis [5].

Research on MMD in Pakistan has been limited, with only a few case reports and case series available in the last two decades and fewer describing the role of EDAS in hemorrhagic MMD [5-7]. Given the prevalence of stroke in young individuals in this region, it raises concerns about the potential underdiagnosis of MMD in Pakistan. This consecutive case series aims to fill the existing gap in the literature by providing a deep insight into the clinical outcomes of EDAS and its safety in MMD. Moreover, it will contribute to developing a standardized treatment approach for patients with MMD in Pakistan by providing data specific to the local population.

## Materials and methods

In this retrospective case series, eight patients of MMD who consecutively underwent EDAS at the Department of Neurosurgery, Punjab Institute of Neurosciences, Lahore, Pakistan, between January 2019 and March 2024 were identified. One patient was excluded owing to non-availability of follow-up data, and hence seven patients were included in this case series. The diagnosis of all these patients was made based on radiological findings from cerebral computed tomography (CT) angiography or magnetic resonance angiography (MRA). The data collection was done retrospectively by reviewing patient data from the hospital database in October 2024. Patient demographics, clinical characteristics, imaging findings, hematological parameters, treatment options, treatment outcomes, and follow-up data were collected in a self-designed questionnaire for easy data collection and interpretation.

The treatment outcomes were recorded after reviewing data from six-month follow-ups in terms of Glasgow coma scale (GCS) score, modified Rankin scale (mRS) score, development of recurrent intracranial hemorrhage in six months, and development of ischemic symptoms (intermittent episodes of headache or any new onset neurological deficit) in six months. Moreover, digital subtraction angiography (DSA) imaging was observed for collateral development according to the Matsushima grading system [8]. This case series was exempted from ethical approval by the local ethical review committee of the hospital.

## Results

A total of seven patients (four males and three females) were included in this study. The mean age of study participants was 14.7±6.8 years. The most common presenting complaint of included patients was an altered state of consciousness secondary to stroke, seen in five (71.4%) patients, followed by headache, seizures, and fever. The motor deficit was the most frequent complaint of patients presenting with hemorrhagic stroke (80%), followed by aphasia and blindness, seen in only 20% of patients presenting with stroke. The past medical history of included patients revealed encephalitis in three (42.8%) patients during the postnatal period, while one (14.2%) patient had a history of cognitive disturbance and aphasia since birth. None of the patients in our case series had any hematological abnormality. Baseline investigations including complete blood count, renal function tests, liver function tests, viral markers, and coagulation profile were normal in all patients. Moreover, the results of cerebrospinal fluid analysis and autoimmune profile were negative for all the included patients. Additionally, three (42.8%) patients reported a history of repeated transient ischemic attacks. The mean preoperative GCS score was 12.8±2.3, and the mean preoperative mRS score was 2.3±1.4. A summary of baseline clinical characteristics of patients included in the case series is given in Table 1.

**Table 1:** Baseline characteristics of study participants ASOC, altered state of consciousness; EDAS, encephaloduroarteriosynangiosis; F, female; GCS, Glasgow coma scale; M, male; mRS, modified Rankin scale; TIA, transient ischemic attack

Patient No.	Age (Years)	Gender (M/F)	Past History	Clinical Presentation	Preoperative GCS Score	Preoperative mRS Score
1	13	M	TIA	ASOC	12	2
2	11	F	Encephalitis	ASOC	11	3
3	12	M	Congenital cognitive disturbance and aphasia	Headache	15	1
4	9	M	Encephalitis	Headache	15	1
5	15	M	Encephalitis	ASOC	13	3
6	18	F	TIA	ASOC, headache, seizures	15	1
7	21	F	TIA	ASOC, fever, fits	9	5

Plain CT of the brain and MRI revealed intracerebral hemorrhage and interventricular hemorrhage in five (71.4%) patients, subarachnoid hemorrhage in three (42.8%) patients, and microbleeds in two (28.5%) patients. Cerebral angiography revealed bilateral occlusion/stenosis of the internal carotid artery, middle cerebral artery, and anterior cerebral artery at various levels with the development of collaterals around the base of the brain including choroidal, lenticulostriate, and thalamic collaterals, while the occurrence of choroidal collaterals in cerebral angiography was a frequent finding in patients presenting with posterior intracerebral hemorrhage.

**Figure 1:**
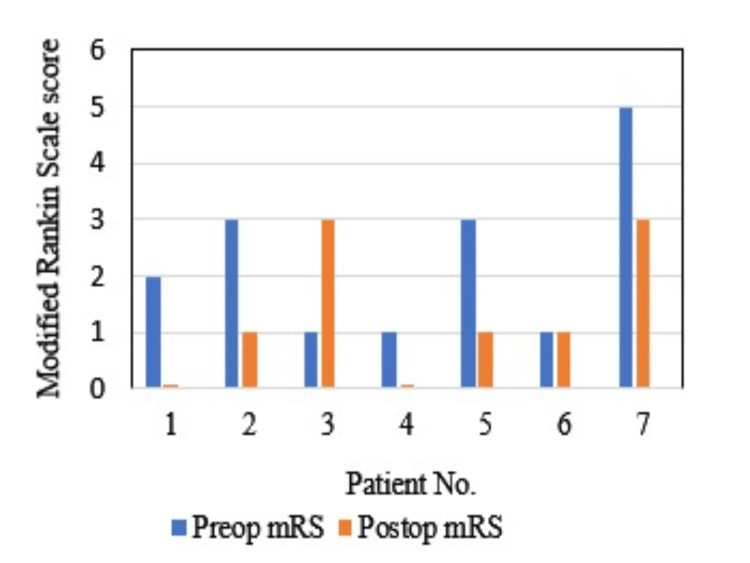
Preoperative versus postoperative mRS scores in study participants mRS, modified Rankin scale

The mean postoperative GCS score was 14.0±1.4, whereas the mean postoperative mRS score was 1.3±1.2. Within-group comparison of mRS scores before and after EDAS revealed improvement in disability score in five (71.4%) patients, no change in one (14.2%) patient, and worsening in one (14.2%) patient (Figure 1).

**Figure 2:**
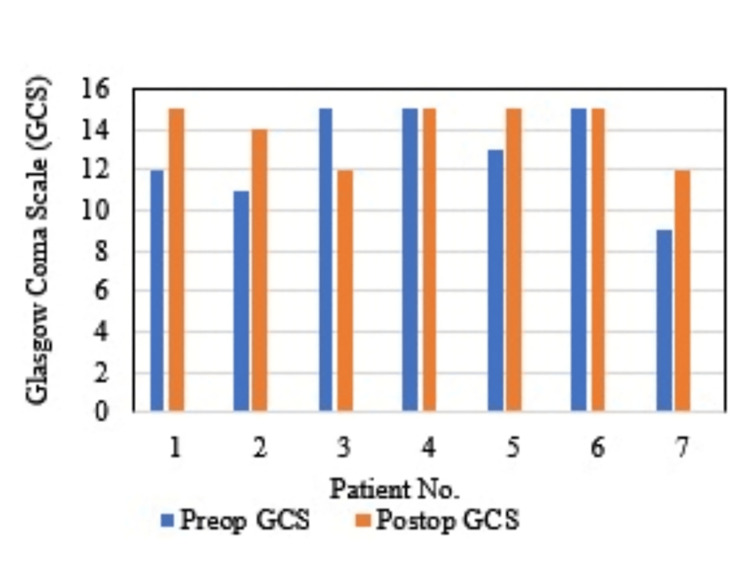
Preoperative versus postoperative GCS scores in study participants GCS, Glasgow coma scale

Similarly, within-group comparison of GCS scores before and after EDAS revealed improvement in four (57.1%) patients, no change in two (28.4%) patients, and worsening in one (14.2%) patient (Figure 2).

Of the seven patients, four (57.1%) patients developed collateral circulation with grade A, whereas three (42.8%) patients had grade B according to the Matsushima grading of collateral development after indirect revascularization. Only one (14.2%) patient in our study deteriorated postoperatively with a deterioration of GCS and mRS scores by more than two points from preoperative levels. None of our patients developed recurrent intracranial hemorrhage postoperatively. Only two patients in our case series reported ischemic symptoms including intermittent headache and new-onset neurological deficit during the six-month follow-up period. The postoperative angiographic findings and surgical outcomes are summarized in Table 2.

**Table 2:** Follow-up angiographic findings and surgical outcomes of EDAS EDAS, encephaloduroarteriosynangiosis; GCS, Glasgow coma scale; mRS, modified Rankin scale

Patient No.	Angiographic Findings	Surgical Outcomes			
	Matsushima grading	Recurrent intracranial hemorrhage	Ischemic symptoms	Postoperative GCS score	mRS score
1	A	None	None	15	0
2	B	None	None	14	1
3	B	None	New-onset neurological deficit	12	3
4	A	None	Intermittent headache	15	0
5	A	None	None	15	1
6	A	None	None	15	1
7	B	None	None	12	3

## Discussion

The term "moyamoya" translates to "a puff of smoke," a name given to the disease by Suzuki and Takaku due to the hazy, “cigarette smoke” appearance of abnormal collaterals on a cerebral angiogram [9].

Our case series report presents the clinical findings of seven patients diagnosed with hemorrhagic MMD who underwent EDAS in our center in the last five years, along with their surgical outcomes in the six-month follow-up period. The sample size of seven for a rare disorder in only five years is quite large in comparison to previous case series from Pakistan reporting 13 patients by retrospectively reviewing data from the last 20 years [7]. This might be due to the fact that the Punjab Institute of Neurosciences is a state-of-the-art center of neurosurgery that provides integrated neuroradiological facilities in Pakistan, catering to patient referrals from all over the country, and the chances of underdiagnosis are comparatively less.

The female preponderance of MMD as described in pre-existing literature is not seen in our study, in which 57.1% of patients were males [10-11]. Additionally, none of our patients showed an autoimmune association with any disease in contrast to a study by Mumtaz et al., who proposed associations of MMD with multiple autoimmune diseases [5].

Intracerebral hemorrhage and interventricular hemorrhage (71.4%) were the most frequently found preoperative radiological findings in our case series, followed by subarachnoid hemorrhage (42.8%) and microbleeds (28.5%). This is further supported in the literature by a recent case report from Pakistan, which highlights the need for considering MMD as a differential diagnosis in adolescents suffering from spontaneous subarachnoid hemorrhage [12].

In our study, out of all the patients who underwent EDAS revascularization, none of the patients experienced a recurrence of intracranial hemorrhage within six months in contrast to 3.3% of recurrent intracranial hemorrhage during the follow-up period after EDAS in a case series presented by Liu et al. [13] and 41.9% occurrence of recurrent intracranial hemorrhage in a longitudinal cross-sectional study by Zhang et al. [14].

In our case series, approximately 72% of patients showed an improvement in functional status in terms of the mRS score. This is similar to the findings of another case series by Rosi et al., who proposed a reduction in the mRS score of patients after EDAS in more than half of the patients. Conversely, the percentage of patients who had worsened mRS scores after EDAS is higher in our study (14.2%) in comparison to the 5.2% reported by Rosi et al. [15].

The majority of patients in our case series had good Matsushima grading (grade A in 57.2% and grade B in 42.8% of patients), while none of the patients had grade C on the Matsushima grading system, even though one patient in our study had a deterioration of functional outcomes in terms of GCS and mRS scores postoperatively. This might be due to the fact provided by Rosi et al., who proposed that the Matsushima grading system had no significant correlation with the clinical outcomes of patients and should not be used as an independent indicator to determine the severity of MMD or success of indirect revascularization technique [15]. Contrarily, Blauwblomme et al. proposed the Matsushima grading system as an important predictor of ischemic recurrence postoperatively after indirect revascularization in MMD [16].

Postoperative symptomatic cerebral ischemia was seen in only two patients in our study, who were 9 and 15 years old. This is supported in the literature by the findings of a study by Choi et al., who proposed that those in the age range of 1 to 15 years were more prone to develop postoperative symptomatic cerebral ischemia, with an overall incidence of 5.1% [17].

This case series highlights the need for considering MMD as an important differential in the pediatric and adolescent population presenting with stroke. Additionally, it demonstrates the role of EDAS in improving functional outcomes of patients with hemorrhagic MMD while ensuring the safety of the procedure. This study provides the audience with future recommendations for conducting longitudinal studies with long-term follow-up at multiple centers to better identify the surgical outcomes of EDAS in MMD.

Limitations

Although our case series highlights the increasing incidence of MMD in Pakistan and favorable surgical outcomes of EDAS in hemorrhagic MMD, the study design, short-term follow-up period, and the single-centered nature of the study limit the generalizability of results.

## Conclusions

MMD is a leading cause of stroke in the pediatric age group. EDAS could be a suitable option for individuals suffering from hemorrhagic MMD despite the risk of ischemic complications. Indirect revascularization via EDAS showed good clinical outcomes in this retrospective cohort study, with no occurrence of recurrent intracranial hemorrhage.
